# Management practices and mortality predictors among *Klebsiella pneumoniae* infections across Lebanese hospitals: a multicenter retrospective study

**DOI:** 10.1186/s12879-025-11010-5

**Published:** 2025-04-28

**Authors:** Rania Itani, Hani M. J. Khojah, Hamza Raychouni, Rahaf Kibrit, Patricia Shuhaiber, Carole Dib, Mariam Hassan, Tareq L. Mukattash, Abdalla El-Lakany

**Affiliations:** 1 Pharmacy Practice Department, Faculty of Pharmacy, Beirut Arab University, Beirut, Lebanon; 2 Department of Pharmacy Practice, College of Pharmacy, Taibah University, Madinah, Kingdom of Saudi Arabia; 3 Intensive Care Unit, Anesthesia Department, Central Military Hospital, Military Healthcare, Lebanese Army, Beirut, Lebanon; 4 Intensive Care Unit, Respiratory Care Department, American University of Beirut Medical Center, Beirut, Lebanon; 5 Intensive Care Unit, Aboujaoudé Hospital, Maten, Lebanon; 6 Pharmacy Department, Lebanese Hospital Geitaoui University Medical Center, Beirut, Lebanon; 7 Pharmacy Department, Mount Lebanon Hospital Balamand University Medical Center, Hazmieh, Lebanon; 8 Emergency Department, Sahel General Hospital, Beirut, Lebanon; 9 Department of Clinical Pharmacy, Faculty of Pharmacy, Jordan University of Science and Technology, Irbid, Jordan; 10 Department of Pharmaceutical Sciences, Faculty of Pharmacy, Beirut Arab University, Beirut, Lebanon; 11 Department of Pharmacognosy, Faculty of Pharmacy, Alexandria University, Alexandria, Egypt

**Keywords:** *Klebsiella pneumoniae*, Antimicrobial drug resistance, ESBL-producing, Carbapenem-resistant, Antibiogram, Antibiotics, Nosocomial infections, Antimicrobial therapy, Mortality predictors, Lebanon

## Abstract

**Background:**

*Klebsiella pneumoniae* is a significant cause of both community-acquired and nosocomial infections, leading to high morbidity and mortality rates. The increasing antimicrobial resistance among *K. pneumoniae* strains poses a critical challenge to effective treatment. This study aimed to assess the appropriateness of initial antimicrobial therapy, determine the 30-day all-cause mortality rate, and identify predictors of mortality among patients infected with *K. pneumoniae* in Lebanese hospitals.

**Methods:**

A multicenter retrospective observational study was conducted across three university hospitals in Beirut, Lebanon. The study included hospitalized adult patients with confirmed *K. pneumoniae* infections. Kaplan-Meier survival analysis and log-rank tests were used to analyze time-to-mortality. Binary logistic regression was performed to identify predictors of mortality.

**Results:**

Of 2,655 cases screened, 410 patients were enrolled, and 395 cases were included in the final analysis of the 30-day mortality after excluding those lost to follow-up. Nearly one-third of the isolates (36.8%) were extended-spectrum β-lactamase (ESBL)-producing, while 6.8% were carbapenem-resistant *K. pneumoniae* (CRKP). The most commonly prescribed empirical antibiotics were meropenem (31.7%), amikacin (28.5%), and ceftriaxone (22.2%). Around one-third of the patients (32.9%) received inappropriate initial antimicrobial therapy. The 30-day mortality rate was 14.4%. Main predictors significantly associated with mortality in patients with *K. pneumoniae* infection were solid cancer (adjusted odds ratio [AOR] = 7.82, *P* < 0.01), coronary artery disease (AOR = 4.81, *P* = 0.01), age ≥ 65 years (AOR = 4.22, *P* = 0.02), type II diabetes mellitus (AOR = 3.96, *P* = 0.01), receiving inappropriate initial antimicrobial therapy (AOR = 2.96, *P* = 0.02), infection with CRKP isolates (AOR = 2.53, *P* = 0.03), and having a higher Charlson comorbidity index (AOR = 1.61, *P* = 0.001).

**Conclusions:**

The study highlights the critical need for effective antimicrobial stewardship and tailored infection control protocols to mitigate the high resistance rates and improve patient outcomes in Lebanon. Emphasis should be placed on enhancing the monitoring of local resistance patterns and using these data to guide the selection of appropriate empirical therapy to reduce mortality associated with *K. pneumoniae* infections.

## Background

*Klebsiella pneumoniae*, a Gram-negative bacterial pathogen, is a common cause for various community-acquired and nosocomial infectious diseases. The global public health impact of *K. pneumoniae* is substantial due to its capacity to induce severe infections associated with high morbidity and mortality rates [1]. This burden is exacerbated by the pathogen’s notable antimicrobial resistance and high propensity to acquire complex resistance traits, which critically limits treatment options [2–4].

*K. pneumoniae* is a member of the ESKAPE group of pathogens, identified by the World Health Organization (WHO) as urgently requiring the development of new antimicrobials due to their critical resistance profiles [5]. The ESKAPE group includes *Enterococcus faecium*, *Staphylococcus aureus*, *K. pneumoniae*, *Acinetobacter baumannii*, *Pseudomonas aeruginosa*, and *Enterobacter spp*. These pathogens demonstrate unique patterns in pathogenesis, transmissibility, and resistance mechanisms posing severe clinical and economic challenges, including increased mortality risk and healthcare costs [6].

In addition, the WHO classifies extended-spectrum β-lactamase (ESBL)-producing Enterobacterales and carbapenem-resistant Enterobacterales (CRE), including *K. pneumoniae*, as critical priority pathogens [5]. This underscores the urgent need for new antibiotics to tackle this significant threat to human health.

The emergence of multidrug-resistant *K. pneumonia* strains, especially ESBL-producing and carbapenem-resistant *K. pneumoniae* (CRKP), poses a considerable challenge due to their potential for community transmission, scarcity of effective treatment, and negative impact on patient outcomes. Previous studies have estimated the pooled 30-day mortality associated with *K. pneumonia* infections to be between 19.3% and 72% [7–12].

Data from a national multicenter study involving 13 Lebanese hospitals during 2015 and 2016, reflecting the country’s antimicrobial susceptibility landscape, reported high resistance of *K. pneumoniae* isolates to various cephalosporin antibiotics [13]. Specifically, resistance to ceftriaxone significantly increased from 35% (2,557 of 7,307 isolates) between 2011 and 2013 to 37% (5,578 of 15,076 isolates) between 2015 and 2016 [13, 14]. Alarmingly, ceftriaxone resistance rates have surged to 63% in 2023. Similarly, resistance to cefuroxime rose from 36 to 39% in the same period, reaching 51.5% from 2021 to 2023. These trends reflect the rapid evolution of resistance among *K. pneumoniae* isolates in Lebanon, likely due to the rising prevalence of ESBL-producing *K. pneumoniae* and CRKP, which confer resistance to various β-lactam antibiotics, including cephalosporins [15–17]. For instance, the prevalence of ESBL ranged between 29.3 and 43% in Lebanese hospitals between 2011 and 2023 [13, 14, 18].

Appropriate initial antimicrobial therapy is essential for managing these infections and improving survival rates. To the best of our knowledge, there is a scarcity of recent studies providing reports on the management practices and the associated overall mortality rates of *K. pneumoniae* infections. Existing studies are mostly restricted to specific types of *K. pneumonia* infections, resistant strains, or specific populations with common underlying medical conditions [7–12].

This study aimed to evaluate the susceptibility patterns of *K. pneumoniae* and assess the appropriateness of the initial antimicrobial therapy (empirical antibiotic therapy) administered to patients infected with this bacterium at three university hospitals in Lebanon. Additionally, it determined the 30-day all-cause mortality rate associated with *K. pneumoniae* infections and identified the predictors associated with mortality in these patients.

## Methods

### Study design, population, and setting

This multicenter, retrospective, and observational study was conducted across three large university hospitals in Beirut, Lebanon. The study reviewed the medical records of hospitalized adult patients (aged 18 years or older) who were infected with *K. pneumoniae* from January 1, 2021, to September 1, 2023. Only the first infection episode was included for patients with multiple infections to capture baseline antimicrobial resistance profiles without the confounding effects of prior treatments or recurrent infections. This approach helped prevent the inflation of resistance rates and overrepresentation of specific resistance profiles, ensuring more accurate and generalizable estimates of *K. pneumoniae* resistance patterns.

Exclusion criteria included incomplete medical records, colonization with *K. pneumoniae* rather than active infection, discharge within 48 h of infection diagnosis, incomplete antimicrobial susceptibility testing (AST) profiles, and polymicrobial infections. To distinguish colonization from active infection, medical records were reviewed based on documented clinical assessments by physicians in progress notes, along with the presence of clinical signs and symptoms consistent with infection. These criteria were established to ensure a homogeneous study population, enhance internal validity, and minimize bias and confounding factors associated with co-infections involving multiple pathogens. This approach enabled a focused analysis of the appropriateness of antimicrobial therapy and the identification of predictors of mortality associated with *K. pneumoniae* infection.

### Microbiological identification

Microbiological identification was carried out at each enrolled hospital following their established protocols. At Hospital 1, bacterial identification and AST were performed using the VITEK 2 system (bioMérieux, Marcy-l’Étoile, France). Meanwhile, Hospitals 2 and 3 employed the disk diffusion method, following the guidelines from the Clinical and Laboratory Standards Institute (CLSI), and interpreted all AST results using CLSI-defined breakpoint values [19].

At Hospital 1, minimum inhibitory concentrations (MICs) were determined using the VITEK 2 system, while Hospitals 2 and 3 measured the diameters of inhibition zones manually, based on the Kirby-Bauer disk diffusion method. The results from the latter hospitals were confirmed with the ADAGIO automated zone reader (Bio-Rad Laboratories, Hercules, CA, USA).

All participating hospitals screened for ESBL production by testing for resistance against third-generation cephalosporins, linsuch as cefotaxime and/or ceftriaxone, in line with CLSI guidelines. CRKP was identified through resistance to imipenem and/or ertapenem, as outlined by the Centers for Disease Control and Prevention (CDC) guidelines [20]. However, confirmatory testing for carbapenemase production was not available at any of the participating centers. For testing colistin susceptibility, Hospital 1 utilized the automated MIC method with VITEK 2, while Hospitals 2 and 3 used the disk diffusion method.

### Development and structure of the data collection form

The principal investigator designed a standardized data collection form for this study following an extensive review of relevant literature [21, 22]. The form was organized into five primary sections, each containing close-ended questions with predefined responses.

### Patient characteristics and sociodemographic information

This section collected data on patients’ demographics and clinical background, including age, sex, admission date, total hospitalization duration, the source of infection acquisition (categorized as either community-acquired or hospital-acquired), as defined in the ***operational definitions section***, recent hospitalization and antibiotic use within the past three month, underlying medical conditions, and the Charlson comorbidity index (CCI).

### Infection onset and pathogen details

This section recorded the onset of infection, primary site of infection, type of specimen obtained, and the antibiogram of the isolated pathogen.

### Empirical therapy information

This section captured detailed information about the empirical therapy administered, including the initiation date relative to the onset of infection, the specific antibiotics used, their routes of administration, dosages, dosage forms, and duration.

The appropriateness of the initiated empirical therapy was assessed by a single researcher with expertise in clinical pharmacy practice in infectious diseases. The assessment followed the definition of appropriate initial antimicrobial therapy outlined in the ***operational definitions*section**. Each case was subsequently validated by the study’s principal investigator, who has expertise in infectious diseases. In instances of disagreement between the researcher and the principal investigator, a consensus was reached through discussion to ensure consistency and reliability in the assessment process.

### De-escalation of therapy

This section focused on whether empirical therapy was adjusted after culture results were available. It included data on the date of de-escalation, the specific antibiotics given as definitive antimicrobial therapy, their doses, and routes of administration.

### Clinical outcomes and associated complications

This section identified the clinical outcomes and associated complications. It evaluated parameters such as survival, 30-day all-cause in-hospital mortality, or loss to follow-up.

A multidisciplinary team comprising clinical pharmacy, epidemiology, and infectious diseases specialists conducted a thorough assessment of the data collection form to ensure its face and content validity. Following this, a pilot test was performed on ten medical records using a convenience sampling approach to evaluate the form’s feasibility and practicality.

### Ethical considerations

The study was conducted in compliance with the World Medical Association Declaration of Helsinki guidelines [23]. It received approval from the institutional review board (IRB) at Beirut Arab University (Approval code: 2023-H-0089-P-R-0532) and from the IRBs and ethical committees of the participating hospitals (Hospital 1: 2023-IRB-012, Hospital 2: HOP-2023-006, Hospital 3: 6/2023). Data collection involved a comprehensive review of de-identified and anonymized patients’ medical records to ensure privacy and confidentiality. The research team did not engage in any direct contact or follow-up with the patients. Given the retrospective and observational nature of the study, informed consent was waived.

### Statistical analysis

The data analysis was performed using the 24th version of the Statistical Package for the Social Sciences (SPSS^®^, IBM Corp., Armonk, NY, USA). Descriptive statistics for categorical variables were presented as frequencies and percentages, while continuous variables were summarized using the mean and standard deviation. Kaplan-Meier survival analysis and the log-rank test were utilized to evaluate time-to-mortality. Univariate analysis was conducted using Pearson’s chi-square test (χ2) to explore associations between independent categorical variables and mortality. The Mann-Whitney U test was applied to assess the association between the CCI and mortality. Variables with a p-value of less than 0.2 in the univariate analysis were included in a logistic regression model for multivariable analysis using a backward selection method. The adjusted odds ratio (AOR) was calculated, and results with a p-value of less than 0.05 and a 95% confidence interval were considered statistically significant.

### Operational definitions

#### Appropriate initial antimicrobial therapy

This parameter evaluated the adequacy of empirically initiated antibiotic therapy. Therapy was considered appropriate if one or more antibiotic agents were administered within 48 h of infection onset, to which the causative pathogen was sensitive according to the AST results. Additionally, the antibiotic had to be delivered via the recommended route and at a dosage regimen appropriate for the patient’s organ function. Empirical antibiotic therapy that did not meet any of these criteria was classified as inappropriate [24].

#### Charlson comorbidity index (CCI)

An extensively validated tool used in clinical research to evaluate comorbidity and predict long-term mortality in patients with multiple underlying medical conditions. The index encompasses sixteen medical conditions, including diabetes, chronic kidney disease, heart failure, and leukemia. Each condition was assigned a score that correlates with the patient’s mortality risk. A higher cumulative score indicated a greater number of comorbidities and an increased risk of death [25–27].

#### Chronic kidney disease

Characterized by an estimated glomerular filtration rate consistently below 60 mL/min/1.73 m² for a period of at least three months prior to hospital admission [28].

#### Carbapenem-resistant *Klebsiella pneumoniae* (CRKP)

Refers to isolates of *K. pneumoniae* that exhibit resistance to imipenem, ertapenem, or meropenem [29].

#### Carbapenem-susceptible *Klebsiella pneumoniae* (CSKP)

Refers to isolates of *K. pneumoniae* that are susceptible to imipenem, ertapenem, and meropenem [29].

#### Community-acquired infection

Defined as an infection that begins before or within 48 h after the patient’s admission to the hospital [30].

#### Definitive antibiotic therapy

Also called targeted antibiotic treatment, involved the administered antibiotic regimen after the causative pathogen had been identified and the results of AST have been obtained [31].

#### Empirical antibiotic therapy

Also known as initial antibiotic therapy, involves the administered antibiotic regimen before the causative pathogen has been identified and before the results of AST have been obtained [22].

#### Multi-drug resistant (MDR)

“acquired non-susceptibility to at least one agent in three or more antimicrobial categories” [32].

#### Nosocomial infection

Also known as hospital-acquired infection, an infection that begins 48 h after the patient’s admission to the hospital [30].

#### Onset of infection

The day when the culture that eventually grew *K. pneumoniae* was collected [22].

#### Previous antibiotic therapy

Having received any systemic antibiotic, including both Gram-positive and Gram-negative antibiotics, for at least 72 h within the three months preceding the onset of infection [28].

#### Primary study outcome

The all-cause mortality within 30 days following the onset of infection [33].

#### Time-to-mortality

The interval between the onset of infection and the occurrence of death [33].

#### Use of antibiotics within the last three months

Receiving any systemic antibiotic for a duration of at least 72 h within the three months preceding the onset of infection [22].

## Results

### Screened and enrolled cases

**Fig. 1:**
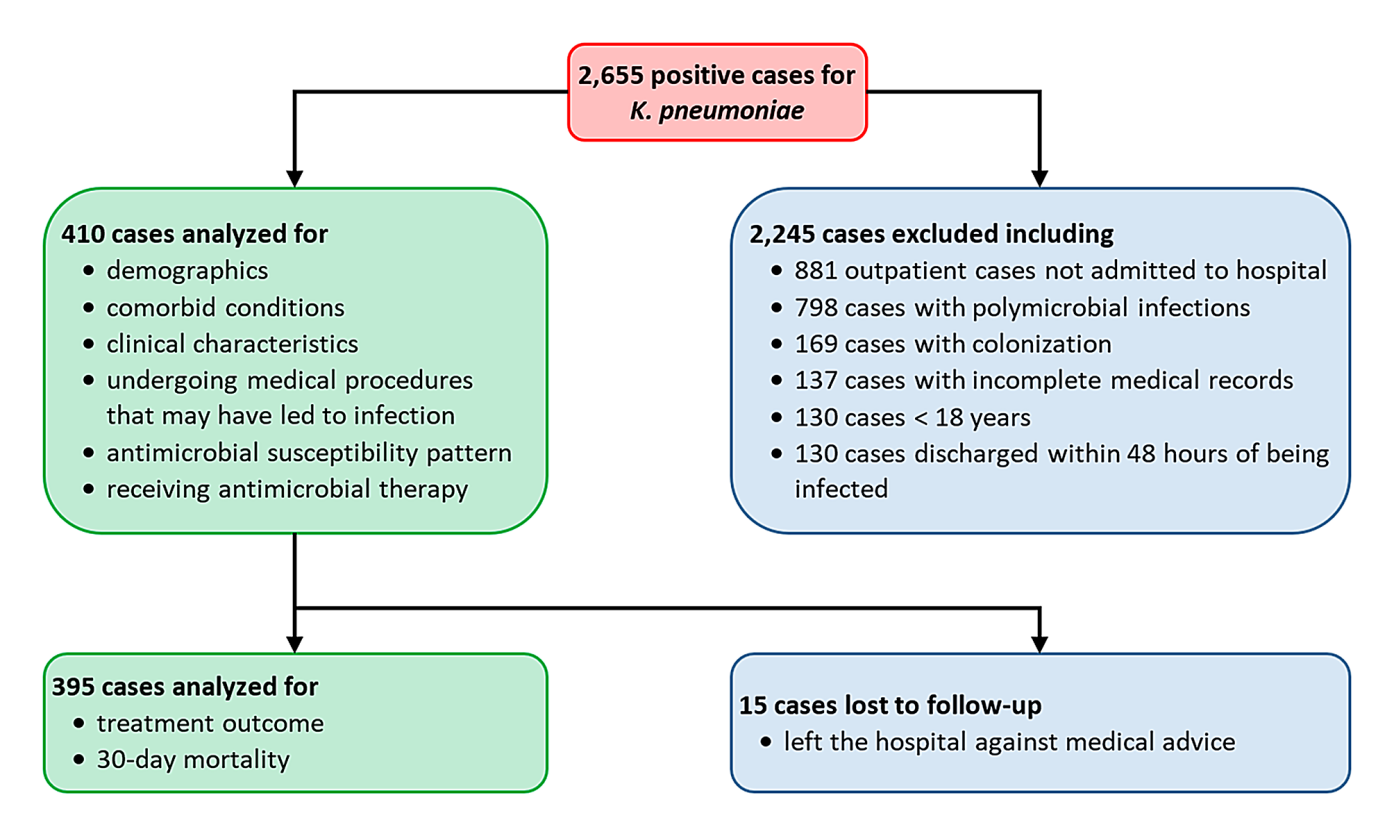
Flowchart of patient enrollment and data analysis for *Klebsiella pneumoniae* infection

A total of 2,655 cases were screened for positive *K. pneumoniae* bacteriologic specimens. Of these, 410 patients met the inclusion criteria and were enrolled in the study. However, only 395 cases were included in the final analysis of treatment outcomes and 30-day mortality, as 15 patients left the hospital against the medical advice and were subsequently lost to follow-up (Fig. 1).

### Patients’ demographic information and clinical characteristics

Table 1 summarizes the sociodemographic information and characteristics of the enrolled patients. The mean age of the patients was 69.58 ± 16.99 years, with an age range of 18 to 98 years, and approximately half were male (215, 52.4%). The mean duration of hospitalization, including the period prior to infection acquiring, was 12.90 ± 9.63 days, with a range of 5 to 89 days. More than half of the infections were community-acquired (232, 56.6%), while the remaining cases were nosocomial, specifically acquired in the internal medicine department (178, 43.4%). A majority of the patients (386, 93.3%) had at least one underlying medical condition, with hypertension (196, 47.8%), type II diabetes mellitus (188, 45.9%), and coronary artery disease (155, 37.8%) being the most prevalent. The mean CCI was 5.17 ± 2.60.

**Table 1:** Demographic and clinical characteristics of patients (*N* = 410)

Characteristics	*n* (%)
**Sex**	
* Male*	215 (52.4)
* Female*	195 (47.6)
**Age** (years)	
* < 45*	41 (10)
* 45–54*	29 (7.1)
* 55–64*	54 (13.2)
* 65–74*	107 (26.1)
* ≥ 75*	179 (43.7)
**Source of infection acquisition**	
***Community-acquired***	232 (56.6)
***Nosocomial (of 178 cases)***	178 (43.4)
Internal medicine	76 (42.6)
Intensive care unit	40 (22.7)
Oncology	22 (12.3)
Cardiac care unit	20 (11.2)
Surgery	20 (11.2)
**Underlying medical conditions** ^*^	
* Alzheimer’s disease*	4 (1)
* Aortic stenosis*	8 (2)
* Atrial fibrillation*	38 (9.3)
* Autoimmune disease*	7 (1.7)
* Benign prostatic hyperplasia*	44 (10.7)
* Chronic kidney disease*	77 (18.8)
* Chronic liver disease*	10 (2.4)
* Chronic obstructive pulmonary disease*	45 (11)
* Congestive heart failure*	71 (17.3)
* Coronary artery disease*	155 (37.8)
* Dementia*	46 (11.2)
* Dyslipidemia*	64 (15.6)
* Epilepsy*	2 (0.5)
* Glaucoma*	4 (1)
* Gout*	6 (1.5)
* History of cerebrovascular accident*	21 (5.1)
* History of trauma*	17 (4.1)
* Hypertension*	196 (47.8)
* Hyperthyroidism*	2 (0.5)
* Hypothyroidism*	22 (5.4)
* Leukemia*	29 (7.1)
* Parkinson’s disease*	4 (1)
* Peptic ulcer disease*	6 (1.5)
* Peripheral vascular disease*	32 (7.8)
* Schizophrenia*	4 (1)
* Solid tumor*	89 (21.7)
* Type II diabetes mellitus*	188 (45.9)
**Use of antibiotics within the last three months**	135 (33)
**Hospitalization within the last three months**	183 (44.6)
**Charlson comorbidity index**	
* Mean ± standard deviation*: * 5.17 ± 2.60 (range: 0–12)*	

^*^ The total number of medical conditions exceeds 410 due to multiple conditions in some patients

### Microbiological characteristics and the antimicrobial susceptibility testing results of *Klebsiella pneumoniae* isolates

The *K. pneumoniae* isolates were collected from various specimen types, including urine (239, 58.3%), skin/wound (51, 12.4%), sputum (39, 9.5%), blood (35, 8.5%), bile fluid (20, 4.9%), deep tracheal aspirate (10, 2.4%), abdominal abscess (6, 1.5%), rectal/perianal swab (6, 1.5%), bronchial aspirate (2, 0.5%), and pleural fluid (2, 0.5%). The sources of infection were identified as urinary tract (239, 58.3%), respiratory tract (51, 12.4%), skin and soft tissue (51, 12.4%), bloodstream (35, 8.5%), intra-abdominal (26, 6.3%), and gastrointestinal tract (6, 1.5%).

Approximately one-third (151, 36.8%) of the *K. pneumoniae* isolates were identified as ESBL-producing, and 28 (6.8%) were CRKP. The AST results of the *K. pneumoniae* isolates are summarized in Table 2. The majority of isolates demonstrated sensitivity to tigecycline (99.4%), imipenem (93.6%), ertapenem (93.1%), amikacin (92.9%), and fosfomycin (91.2%). Additionally, a substantial proportion of isolates were sensitive to norfloxacin (77.3%), prulifloxacin (75.2%), piperacillin/tazobactam (71.8%), and ciprofloxacin (71.4%). Conversely, approximately half of the isolates exhibited resistance to cephalosporins, including ceftriaxone (63%), cefuroxime (51.5%), cefixime (51.2%), and ceftazidime (48.3%).

**Table 2:** In vitro antimicrobial susceptibility testing results of *Klebsiella pneumoniae* isolates (*N* = 410)

Antibiotic Class	Antibiotic	Isolates^a^ (*n*)	Sensitive *n* (%)	Resistant *n* (%)	Intermediate *n* (%)
Penicillins	Ampicillin	402	1 (0.2)	401 (99.8)	0 (0)
	Amoxicillin/clavulanic acid	390	170 (43.6)	192 (49.2)	28 (7.2)
	Piperacillin	194	30 (15.5)	154 (79.4)	10 (5.2)
	Piperacillin/tazobactam	390	280 (71.8)	93 (23.8)	17 (4.4)
Cephalosporins	Cephalexin	11	5 (45.5)	5 (45.5)	1 (9.1)
	Cefazolin	38	22 (57.9)	15 (39.5)	1 (2.6)
	Cefepime	397	222 (55.9)	174 (43.8)	1 (0.3)
	Cefixime	328	160 (48.8)	168 (51.2)	0 (0)
	Cefotaxime	389	192 (49.4)	194 (49.9)	3 (0.8)
	Ceftriaxone	100	37 (37)	63 (63)	0 (0)
	Cefuroxime	404	196 (48.5)	208 (51.5)	0 (0)
	Cefoxitin	375	307 (81.9)	68 (18.1)	0 (0)
	Ceftaroline	17	11 (64.7)	6 (35.3)	0 (0)
	Ceftazidime	400	200 (50)	193 (48.3)	7 (1.8)
Monobactam	Aztreonam	348	194 (55.7)	152 (43.7)	2 (0.6)
Carbapenems	Ertapenem	408	380 (93.1)	28 (6.9)	0 (0)
	Imipenem	406	380 (93.6)	24 (5.9)	2 (0.5)
Aminoglycosides	Amikacin	396	368 (92.9)	22 (5.6)	6 (1.5)
	Gentamicin	400	327 (81.8)	71 (17.8)	2 (0.5)
	Tobramycin	60	49 (81.7)	9 (15)	2 (3.3)
Quinolones	Ciprofloxacin	402	287 (71.4)	113 (28.1)	2 (0.5)
	Norfloxacin	198	153 (77.3)	45 (22.7)	0 (0)
	Prulifloxacin	266	200 (75.2)	66 (24.8)	0 (0)
Polymyxins	Colistin	26	24 (92.3)	2 (7.7)	0 (0)
Tetracyclines	Tetracycline	278	178 (64)	100 (36)	0 (0)
Glycylcyclines	Tigecycline	342	340 (99.4)	2 (0.6)	0 (0)
Nitrofurans	Nitrofurantoin	270	65 (24.1)	157 (58.1)	48 (17.8)
Fosfomycin	Fosfomycin	182	166 (91.2)	12 (6.8)	4 (2.2)
Trimethoprim	Trimethoprim/sulfamethoxazole	366	213 (58.2)	153 (41.8)	0 (0)

^a^ Number of isolates tested with each antibiotic

### Assessment of antimicrobial therapy appropriateness

Among the 410 patients included in the study, nearly half (206, 50.2%) received combination empirical antimicrobial therapy, with almost all of these antimicrobials being administered intravenously (404, 98.5%), as shown in Table 3. The most commonly prescribed agents were meropenem (130, 31.7%), amikacin (117, 28.5%), and ceftriaxone (91, 22.2%).

Approximately one-third of the patients (135, 32.9%) received inappropriate initial antimicrobial therapy. Of the 410 patients, 70 (17.1%) were initially prescribed antibiotics to which the causative pathogen was resistant, based on susceptibility tests. Additionally, 6 patients (1.5%) experienced a delay in receiving the therapy, with administration occurring 48 h after the onset of infection. Moreover, 74 patients (18%) received inappropriate doses of the initial antibiotics. Despite these issues, all patients received the initial antimicrobial therapy via the appropriate route of administration.

**Table 3:** Empirical antimicrobial therapy administered (*N* = 410)

Information	*n* (%)
**Onset of initiation with respect to the onset of infection**	
* Mean = 1.2 ± 0.76 days (range = 1–7)*	404 (98.5)
**Route of administration**	
* Intravenous*	404 (98.5)
* Oral*	6 (1.5)
**Monotherapy vs. combination therapy**	
* Monotherapy*	204 (49.7)
* Combination*	206 (50.2)
**Antimicrobial agents used (*N*** = 410)^*^	
* Meropenem*	130 (31.7)
* Amikacin*	117 (28.5)
* Ceftriaxone*	91 (22.2)
* Piperacillin/tazobactam*	82 (20)
* Imipenem/cilastatin*	46 (11.2)
* Vancomycin*	42 (10.2)
* Levofloxacin*	28 (6.8)
* Metronidazole*	25 (6.1)
* Teicoplanin*	22 (5.4)
* Ciprofloxacin*	20 (4.9)
* Clindamycin*	10 (2.4)
* Cefotaxime*	8 (2)
* Trimethoprim/sulfamethoxazole*	8 (2)
* Ceftazidime/avibactam*	6 (1.5)
* Azithromycin*	6 (1.5)
* Colistin*	6 (1.5)
* Cefepime*	4 (1)
* Tigecycline*	4 (1)
* Ertapenem*	3 (0.7)
* Cefixime*	2 (0.5)
* Clarithromycin*	2 (0.5)
* Amoxicillin/clavulanic acid*	2 (0.5)
* Doxycycline*	2 (0.5)
* Linezolid*	2 (0.5)
**Commonly prescribed combination therapies (*n*** **= 206)**	
* Ceftriaxone + amikacin*	29 (14)
* Meropenem + amikacin*	27 (13)
* Meropenem + vancomycin + amikacin*	18 (8.7)
* Piperacillin/tazobactam + vancomycin + amikacin*	10 (4.8)
* Piperacillin/tazobactam + amikacin*	6 (2.9)
* Meropenem + vancomycin*	6 (2.9)
* Azithromycin + ceftriaxone*	5 (2.4)
**Assessment of appropriateness (*N*** **= 410)**	
***Sensitive to the culture results***	
No	70 (17.1)
Yes	340 (82.9)
***Initiated within 48 h of the onset of infection***	
No	6 (1.5)
Yes	404 (98.5)
***Given for at least 48 h***	
No	0 (0)
Yes	410 (100)
***Given with an appropriate dose***	
No	74 (18)
Yes	336 (82)
***Given through an appropriate route of administration***	
No	0 (0)
Yes	410 (100)
***Overall appropriateness***	
No	135 (32.9)
Yes	275 (67.1)

^*^ As multiple responses were recorded, numbers do not add up to 410

Notably, 15 patients (3.7%) died while receiving empirical antimicrobial therapy, and another 15 patients (3.7%) left the hospital against medical advice, resulting in a loss to follow-up. Among the remaining survivors (*n* = 380), 235 (61.9%) were transitioned to definitive antibiotic therapy, whereas 145 patients (38.1%) continued with empirical antimicrobial therapy even after obtaining culture results. Of the patients initially receiving combination therapy, 78.8% underwent therapy modification, including shifting or de-escalation, during their treatment course.

The mean time to initiate definitive antimicrobial therapy based on culture results was 2.37 ± 1.88 days. The most commonly administered definitive antibiotic was meropenem (120, 26.8%), followed by ceftriaxone (63, 16.6%) and ciprofloxacin (54, 14.2%). Importantly, more than half of the patients (202, 53.1%) received definitive antimicrobial therapy that did not correspond to the narrowest sensitive antibiotics as per the antbiogram results. For further details on definitive antimicrobial therapy, please refer to Table 4.

**Table 4:** Definitive antimicrobial therapy administered (*N* = 380)^a^

Information	*n* (%)
**Therapy was shifted or de-escalated to definitive antimicrobial therapy**	
* No*,* kept on empirical therapy*	145 (38.1)
* Yes*	235 (61.9)
**Onset of initiation relative to obtaining the culture results**	
* Mean = 2.37 ± 1.88 days (range = 1–11)*	
**Route of administration**	
* Intravenous*	302 (79.5)
* Oral*	78 (20.5)
**Monotherapy vs. combination therapy**	
* Monotherapy*	303 (79.8)
* Combination*	77 (20.2)
**Antimicrobial agents used** ^**b, c**^	
* Meropenem*	102 (26.8)
* Ceftriaxone*	63 (16.6)
* Ciprofloxacin*	54 (14.2)
* Imipenem/cilastatin*	42 (11.1)
* Piperacillin/tazobactam*	36 (9.5)
* Amikacin*	24 (6.3)
* Cefixime*	22 (5.8)
* Metronidazole*	16 (4.2)
* Levofloxacin*	14 (3.7)
* Tigecycline*	14 (3.7)
* Colistin*	12 (3.2)
* Ertapenem*	12 (3.2)
* Teicoplanin*	10 (2.6)
* Vancomycin*	8 (2.1)
* Ceftazidime/avibactam*	7 (1.8)
* Amoxicillin/clavulanic acid*	4 (1.1)
* Trimethoprim/sulfamethoxazole*	4 (1.1)
* Fosfomycin*	4 (1.1)
* Cefuroxime*	2 (0.5)
* Cefepime*	2 (0.5)
* Cefotaxime*	2 (0.5)
* Ceftizoxime*	2 (0.5)
* Nitrofurantoin*	2 (0.5)
**Assessment of appropriateness**	
***Sensitive to the culture results***	
No	24 (6.3)
Yes	356 (93.7)
***Prompt de-escalation post obtaining the culture results (within 24 h)***	
No, delayed de-escalation/shifting to definitive therapy	67 (17.6)
Yes	168 (44.2)
Kept on empirical antimicrobial therapy	145 (38.2)
***Given with an appropriate dose***	
No	40 (10.5)
Yes	340 (89.5)
***The selected antibiotic had the narrowest spectrum according to the antibiogram***	
No, there were narrower alternatives that the isolated pathogen was also sensitive to	202 (53.1)
Yes	178 (46.8)
***The selected antibiotic was safe according to drug and patient-specific factors***	
No, there were safer alternatives	117 (30.8)
No, but there were no safer alternatives	36 (9.5)
Yes	227 (59.7)

^a^ Thirty patients out of the initial 410 died or were lost to follow-up during the empirical therapy

^b^ As multiple responses were recorded, numbers do not add up to 380

^c^ Listed antimicrobial agents represent both continued and newly initiated therapies

### Clinical outcomes and complications associated with *Klebsiella pneumoniae* infections

Excluding the 15 patients who were lost to follow-up after leaving the hospital against medical advice, a total of 395 cases were analyzed. Among these, 57 patients (14.4%) died within 30 days of infection onset during their hospital stay, including the 15 who died during empirical therapy. The mean time to mortality was 10.23 ± 6.57 days.

A significant proportion of the analyzed cases (173, 43.8%) developed sepsis or septic shock following *K. pneumoniae* infection. The mean sequential organ failure assessment (SOFA) score was 8.43 ± 2.87 (range: 5 to 13), while the mean quick SOFA score was 1.66 ± 0.99 (range: 0 to 3).

Notably, almost one-quarter of the patients experienced acute kidney injury (94, 23.8%) and pleural effusion (90, 22.8%). Other complications associated with *K. pneumoniae* infections included atelectasis (28, 7.1%), thrombocytopenia (24, 6.1%), neutropenia (22, 5.6%), acute respiratory distress syndrome (14, 3.5%), acid-base imbalances (14, 3.5%), disseminated intravascular coagulation (12, 3.0%), and peripheral necrosis following skin infection leading to amputation (6, 1.5%).

### Comparison of survival of patients with *Klebsiella pneumoniae* infections according to the appropriateness of initial antimicrobial therapy

**Fig. 2:**
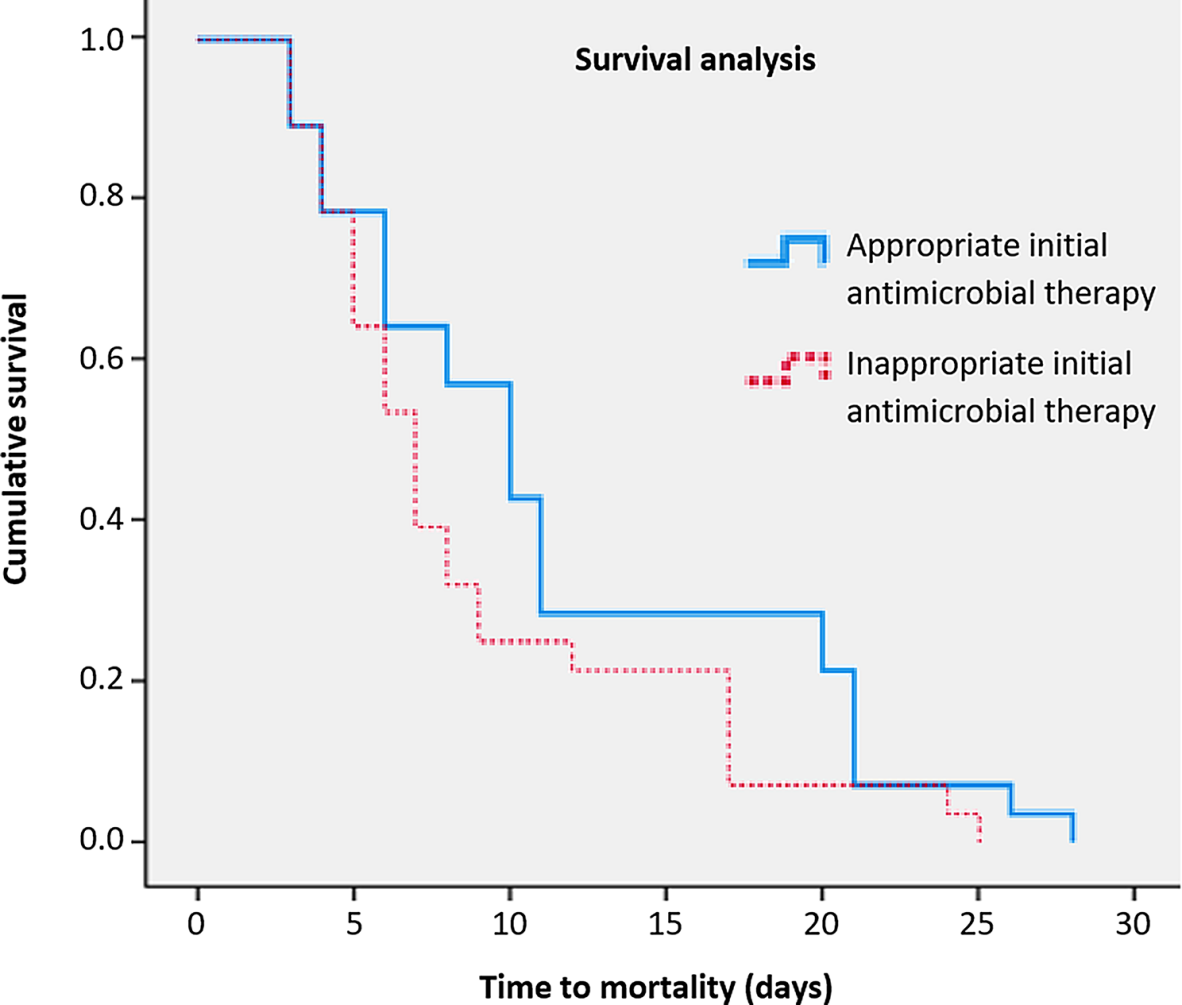
Kaplan-Meier survival curves for patients with *Klebsiella pneumoniae* infection by initial antimicrobial therapy appropriateness

Figure 2 shows that the Kaplan-Meier survival curve for patients with *K. pneumoniae* infections revealed a non-significant shorter time to mortality for those receiving inappropriate versus appropriate initial antimicrobial therapy (28 vs. 57, log-rank test = 11.49, *P* = 0.08). The mean time to death was 8.92 ± 6.20 days for inappropriate therapy and 11.53 ± 7.52 days for appropriate ‏therapy.

### Predictors of mortality in patients with *Klebsiella pneumoniae* infection

Univariate analysis identified a significant association between respiratory tract infection and mortality (unadjusted odds ratio [UOR] = 3.50, 95% CI = 1.70–7.18, *P* < 0.001) (Table 5). Further analysis using binary logistic regression with backward stepwise method revealed nine predictors significantly associated with mortality in patients infected with *K. pneumoniae* (Table 6).

The results indicated that patients aged ≥ 65 years (AOR = 4.22, 95% CI = 1.20–14.80, *P* = 0.02), those with nosocomial infections (AOR = 6.66, 95% CI = 2.68–10.5, *P* < 0.001), type II diabetes mellitus (AOR = 3.96, 95% CI = 1.77–8.86, *P* = 0.01), coronary artery disease (AOR = 4.81, 95% CI = 1.96–11.85, *P* = 0.01), and solid cancer (AOR = 7.82, 95% CI = 2.71–12.5, *P* < 0.01) had a higher likelihood of mortality. Additionally, a higher CCI was significantly associated with mortality (AOR = 1.61, 95% CI = 1.12–5.9, *P* = 0.001).

Moreover, infections with CRKP isolates (AOR = 2.53, 95% CI = 0.84–7.63, *P* = 0.03), and receiving initial inappropriate antimicrobial therapy (AOR = 2.96, 95% CI = 0.66–3.26, *P* = 0.02) were also significantly linked to increased mortality.

**Table 5:** Univariate analysis of predictors associated with mortality in patients with *Klebsiella pneumoniae* infection (*N* = 395)^a^

Characteristics	*n* (%)^b^	30-Day all-cause mortality		UOR (95% CI)	Pearson’s 𝑋^2^	*P* ^d^
		Survived (338) *n* (%)^c^	Died (57) *n* (%)^c^			
**Age group** (reference: 18–64 years)						
18–64	118 (29.9)	107 (90.7)	11 (9.3)	-	4.76	0.03^e^
≥ 65	277 (70.1)	231 (83.3)	46 (16.7)	1.94 (0.97–3.89)		
**Sex** (reference: female)						
Male	208 (52.7)	181 (87)	27 (13)	0.78 (0.44–1.37)	0.74	0.38
Female	187 (47.3)	157 (84)	30 (16)	-		
**Source of infection acquisition** (reference: community-acquired)						
Community-acquired	220 (55.7)	207 (94.1)	13 (5.9)	-	29.20	< 0.001^e^
Nosocomial	175 (44.3)	131 (74.9)	44 (25.1)	5.35 (2.77–10.31)		
**Hospital department of infection acquisition** (reference: internal medicine)						
Internal medicine	73 (41.7)	66 (90.4)	7 (9.6)	-	47.72	< 0.001^e, f^
Intensive care unit	40 (22.8)	23 (57.5)	17 (42.5)	6.97 (2.56–18.94)		
Oncology	22 (12.5)	14 (63.6)	8 (36.4)	5.39 (1.68–17.31)		
Cardiac care unit	20 (11.5)	14 (70)	6 (30)	4.04 (1.18–13.87)		
Surgery	20 (11.5)	18 (90)	2 (10)	1.05 (0.50–5.49)		
**Underlying medical conditions** (reference: no history of the related condition)						
Hypertension	185 (46.8)	158 (85.4)	27 (14.6)	1.03 (0.58–1.80)	0.08	0.93
Type II diabetes mellitus	180 (45.6)	147 (81.7)	33 (18.3)	1.79 (1.01–3.15)	4.08	0.04^e^
Coronary artery disease	153 (38.7)	122 (79.7)	31 (20.3)	2.11 (1.20–3.72)	6.87	< 0.01^e^
Solid tumor	87 (22)	67 (77)	20 (23)	2.19 (1.19–4.01)	6.61	0.01^e^
Chronic kidney disease	74 (18.7)	63 (85.1)	11 (14.9)	1.04 (0.51–2.13)	0.01	0.90
Congestive heart failure	69 (17.5)	51 (73.9)	18 (26.1)	2.60 (1.38–4.89)	9.20	0.002^e^
Dyslipidemia	62 (15.7)	54 (87.1)	8 (12.9)	0.86 (0.39–1.91)	0.13	0.70
Benign prostatic hyperplasia	40 (10.1)	36 (90)	4 (10)	0.63 (0.22–1.85)	0.70	0.40
Chronic obstructive lung disease	45 (11.4)	33 (73.3)	12 (26.7)	2.46 (1.19–5.12)	6.15	0.01^e^
Atrial fibrillation	36 (9.1)	26 (72.2)	10 (27.8)	2.55 (1.16–5.63)	5.71	0.01^e^
**Previous hospitalization within the last 3 months** (reference: no)						
No	220 (55.7)	190 (86.4)	30 (13.6)	-	0.25	0.61
Yes	175 (44.3)	148 (84.6)	27 (15.4)	1.16 (0.66–2.03)		
**Previous antibiotics use within the last 3 months** (reference: no)						
No	267 (67.6)	239 (89.5)	28 (10.5)	-	10.37	0.001^e^
Yes	128 (32.4)	99 (77.3)	29 (22.7)	2.50 (1.41–4.42)		
**Carbapenem-resistant*Klebsiella pneumoniae*** (reference: CSKP)						
CSKP	367 (93)	321 (87.4)	46 (12.6)	-	13.01	< 0.001^e^
CRKP	28 (7)	17 (60.7)	11 (39.3)	4.52 (1.99–10.24)		
**Extended-spectrum β-lactamase (ESBL)-producing*Klebsiella pneumoniae*** (reference: no)						
No	245 (62)	217 (88.6)	28 (11.4)	-	4.71	0.03^e^
Yes	150 (38)	121 (80.7)	29 (19.3)	1.86 (1.06–3.27)		
**Source of infection** (reference: urinary tract)						
Urinary tract	225 (57)	199 (88.4)	26 (11.6)	-	16.23	< 0.001^e, h^
Respiratory tract	51 (12.9)	35 (68.6)	16 (31.4)	3.50 (1.70–7.18)		
Soft tissue/wound	50 (12.6)	41 (82)	9 (18)	1.68 (0.73–3.85)		
Bloodstream	37 (9.4)	33 (89.2)	4 (10.8)	0.93 (0.30–2.83)		
Intra–abdominal	26 (6.6)	24 (92.3)	2 (7.7)	0.64 (0.14–2.86)		
Gastrointestinal	6 (1.5)	6 (100)	0 (0)	-		
**Appropriateness of the initial antimicrobial therapy** (reference: appropriate)						
Inappropriate	135 (34.2)	107 (79.3)	28 (20.7)	2.08 (1.18–3.68)	6.61	0.01^e^
Appropriate	260 (65.8)	231 (88.8)	29 (11.2)	-		
**Charlson comorbidity index**						
Mean ± SD	5.21 ± 2.58	5.09 ± 2.63	7.91 ± 2.20	-	-	0.02^g^

***CRKP***, carbapenem-resistant *Klebsiella pneumoniae*; ***CSKP***, carbapenem-sensitive *Klebsiella pneumoniae*; ***ESBL***, extended-spectrumβ-lactamase-*Klebsiellapneumoniae*; ***SD***, standard deviation; ***UOR***, unadjusted odds ratio

^a^ Fifteen patients out of the enrolled 410 left the hospital against medical advice (lost to follow-up)

^b^ Percentages for the column

^c^ Percentages for the row

^d^ Univariate analysis was conducted to test the associations between variables with mortality

^e^ Statistically significant (𝑃 < 0.05)

^f^ The only significant difference was observed between the intensive care unit and all other groups

^g^ Mann-Whitney U test was conducted to test the association between Charlson comorbidity index with mortality

^h^ The only significant difference was observed between the respiratory tract and all other groups

**Table 6:** Logistic regression analysis^a^ of the significant predictors associated with mortality in patients with *Klebsiella pneumoniae* infection

Predictors	UOR	B	SE	Wald	AOR	95% CI	*P*
**Constant**	-	-6.41	0.92	48.5	0.02	-	< 0.01
**Age** (reference: 18–64 years)							
≥ 65	1.94	1.44	0.64	5.06	4.22	1.20–14.80	0.02^b^
**Community-acquired vs. nosocomial** (reference: community-acquired)							
Nosocomial infection	5.35	1.89	0.46	16.75	6.66	2.68–10.5	< 0.001^b^
**Type II diabetes mellitus** (reference: no)							
Yes	1.79	1.37	0.41	11.28	3.96	1.77–8.86	0.01^b^
**Coronary artery disease** (reference: no)							
Yes	2.11	1.57	0.46	11.68	4.81	1.95–11.85	0.01^b^
**Solid cancer** (reference: no)							
Yes	2.19	2.05	0.54	14.54	7.82	2.71–12.5	< 0.001^b^
**Carbapenem-resistant*Klebsiella pneumoniae*** (reference: no)							
Yes	4.28	0.93	0.56	2.73	2.53	0.84–7.63	0.03^b^
**Extended-spectrum β-lactamase producing*Klebsiella pneumoniae*** (reference: no)							
Yes	1.86	0.35	0.46	0.73	1.48	0.60–3.66	0.39
**Appropriateness of the initial antimicrobial therapy** (reference: appropriate)							
Inappropriate	2.08	0.38	0.40	0.89	2.96	0.66–3.26	0.02^b^
**Charlson comorbidity index**	-	0.46	0.14	10.33	1.62	1.12–5.9	0.001^b^

***AOR***, adjusted odds ratio; ***B***, coefficient for the constant in the null model; ***CI***, confidence interval; **ESBL**, extended-spectrum β-lactamase-*Klebsiella pneumoniae*; ***SE***, standard error; ***UOR***, unadjusted odds ratio; ***Wald***, Wald chi-square test that tests the null hypothesis

^a^ Binary logistic regression using backward stepwise analysis

^b^ Statistically significant (𝑃 < 0.05)

## Discussion

This study demonstrates high resistance among *K. pneumoniae* isolates to various cephalosporin antibiotics, with almost half exhibiting resistance to second-, third-, and fourth-generation cephalosporins. These findings align with growing concerns about the declining efficacy of cephalosporins in treating severe infections caused by *K. pneumoniae*, particularly in hospitalized patients. The historical data from Lebanon indicate an increase in CRKP prevalence from 0% in 2008 to 4% in 2014 [34]. The current study’s rate of 6.8% suggests continuing upward trend, emphasizing the urgency of re-evaluating cephalosporins as viable options for empirical therapy in serious *K. pneumoniae* infections in hospitalized patients.

Carbapenems have emerged as the preferred antibiotic class for empirical treatment of *K. pneumoniae* infections, with this study reporting sensitivity rates exceeding 93%. This finding is consistent with national studies, conducted over the past decade, which documented *K. pneumoniae* susceptibility rates to carbapenems ranging from 93.5 to 98.5% between 2008 and 2018 [13, 14, 34–36]. However, regional disparities in CRKP prevalence are notable. For example, a recent study in Jordan reported a prevalence of 26% [37], while Iran reported a prevalence of 25%, with 13.0% of isolates resistant to both imipenem and meropenem [38]. In Saudi Arabia, resistance rates were even higher, with 57.4% and 63% of isolates resistant to meropenem and imipenem, respectively, according to a 2015 observational study [39]. These variations in CRKP prevalence can be attributed to multiple factors, including differences in healthcare infrastructure, antibiotic prescription practices, patient demographics, and regional antimicrobial stewardship initiatives.

Fosfomycin, an older bactericidal antibiotic, showed promising sensitivity rates in this study, making it a potential option for treating MDR infections. This high sensitivity is likely due to its limited use, particularly in hospital settings [40]. In Lebanon, fosfomycin is available only as an oral formulation, mainly prescribed for acute cystitis [41] and occasionally used off-label for prostatitis [42]. In contrast, intravenous (IV) formulations available in other countries have gained renewed attention as effective agents against MDR pathogens, including ESBL-producing *K. pneumoniae* [40, 42, 43]. Given its favorable microbiological profile and safety record, fosfomycin could be a viable alternative for empiric therapy [40, 42, 43]. However, its broader adoption in Lebanon requires the availability of IV formulations. Policymakers should consider introducing IV fosfomycin to the local market to expand treatment options. Additionally, future randomized controlled trials are needed to confirm its efficacy and safety in treating a wider range of MDR infections.

Empirical antibiotic therapy plays a vital role in managing bacterial infections, particularly when timely administration is critical to patient outcomes. Therapy must begin immediately after obtaining diagnostic specimens, guided by factors such as the infection site, severity, patient comorbidities, and resistance patterns. Patient-specific considerations, including renal and hepatic function, immunologic status, pregnancy/lactation, and recent antimicrobial exposure, are essential for tailoring therapy [31, 44, 45]. Empirical therapy should also adhere to established guidelines and reflect local resistance patterns to ensure optimal outcomes [31, 44–46]. Once pathogens are identified through cultures and/or AST results, therapy should transition to definitive treatments targeting the causative organism. This approach reduces unnecessary exposure to broad-spectrum agents, minimizes toxicity, and curtails resistance development [31, 44–47].

In this study, approximately one-third of patients received inappropriate initial antimicrobial therapy, often involving antibiotics such as ceftriaxone, piperacillin/tazobactam, and levofloxacin, which showed limited susceptibility. Local resistance rates exceeding 20% typically render antibiotics unsuitable for empirical use, highlighting the importance of selecting agents with proven efficacy in the local context [48–51]. Carbapenems and aminoglycosides remain preferred options for severe *K. pneumoniae* infections, particularly in hospitalized patients at high risk for ESBL-producing organisms [52, 53]. Their lower resistance rates and higher efficacy make them essential tools in the fight against MDR infections.

This study’s reported 30-day all-cause mortality rate of 14.4% is noteworthy. While lower than the 19.3% reported in a Taiwanese study on *K. pneumoniae* bacteremia [9] or the pooled 29% mortality rate from a systematic review [10], it reflects significant clinical challenges. In addition, a recent study conducted in a tertiary care center in Palestine reported a crude mortality rate of 17.9% associated with CREs including *K. pneumoniae* [54]. Variations in mortality rates across studies can result from differences in patient populations, healthcare practices, and infection management protocols. For instance, timely interventions such as surgical debridement, abscess drainage, and catheter removal often influence outcomes. Factors significantly associated with higher mortality in this study included advanced age, nosocomial infections, high CCI scores, infection with CRKP isolates, and inappropriate initial therapy. These findings are consistent with previous research linking comorbidities such as diabetes, coronary artery disease, and malignancies to increased mortality risks [9, 55]. A higher CCI is associated with an elevated risk of mortality due to longer hospital stays and increased susceptibility to severe infections due to patients’ weakened immune function [56].

CRKP infections pose a particularly severe challenge. Systematic reviews have shown mortality rates of 42.1% for CRKP infections compared to 21.6% for CSKP infections [21, 55, 57]. The increased mortality associated with CRKP can be attributed to its extensive resistance profile, which often includes resistance to β-lactam antibiotics, aminoglycosides, and fluoroquinolones. Additionally, CRKP’s ability to persist and spread within hospital environments exacerbates its impact, leading to nosocomial outbreaks and further complicating treatment efforts [55, 57]. These findings underscore the importance of robust infection control measures and the implementation of targeted antimicrobial stewardship programs to limit CRKP transmission and improve patient outcomes.

Inappropriate initial antimicrobial therapy was found to triple the risk of mortality in this study, highlighting the critical need for timely and effective empirical treatments. Empirical agents must provide adequate in vitro activity against the most likely pathogens, as delays in initiating appropriate therapy significantly reduce survival rates [58–62]. To address these issues, healthcare providers must prioritize the use of evidence-based protocols, strengthen antimicrobial stewardship programs, and develop robust surveillance systems to monitor resistance patterns.

The findings of this study highlight an urgent need for national action to combat antimicrobial resistance in Lebanon. Key priorities include the establishment of surveillance systems to track resistance trends, the implementation of infection control protocols, and the development of national treatment guidelines. Policymakers should also consider expanding access to effective agents, such as IV fosfomycin, to enhance treatment options for MDR infections. These measures are essential for mitigating the impact of antimicrobial resistance, improving clinical outcomes, and ensuring the long-term efficacy of available treatments.

### Study limitations

The study focused exclusively on hospitalized patients, likely overestimating resistance patterns and limiting the generalizability of findings to community settings. Moreover, only first infection episodes were analyzed, excluding follow-up infections that could have provided insights into disease progression and antimicrobial resistance development. Additionally, the exclusion of polymicrobial infections, despite their prevalence in clinical practice, further reduced the applicability of the findings to real-world scenarios. Another limitation was that variability in AST across the three laboratories may have influenced resistance data and reduced comparability between centers. Furthermore, assessment of initial antimicrobial therapy relied on individual evaluations, potentially introducing bias, and lacked external validation. At the same time, empirical therapy evaluation did not account for resistance mechanisms such as ESBL or AmpC production, while extrapolated susceptibility data for untested antibiotics may have reduced clinical relevance. In addition, definitive antimicrobial therapy was not comprehensively assessed, omitting critical factors such as drug selection, dosage, and therapy duration. The study was also limited by the fact that its scope was restricted to three academic hospitals in Beirut over three years, which may not fully represent resistance trends or management practices. Moreover, complications could not be definitively attributed to *K. pneumoniae* infections, as other factors like adverse drug reactions or comorbidities may have contributed. Finally, the study’s primary outcome, 30-day in-hospital mortality, excluded post-discharge mortality, readmissions, and follow-up cultures, restricting the understanding of long-term impacts and infection-specific mortality.

To address these limitations, future large-scale, longitudinal studies conducted across diverse Lebanese hospitals are needed to provide a more comprehensive understanding of resistance patterns, treatment practices, and patient outcomes nationwide.

## Data Availability

The dataset discussed in this article is accessible only upon reasonable request due to the presence of confidential information. To request access to the datasets, please contact the first author at r.itani@bau.edu.lb.
